# Lay-Led Intervention for War and Refugee Trauma: A Randomized Clinical Trial

**DOI:** 10.1001/jamanetworkopen.2024.29661

**Published:** 2024-08-26

**Authors:** Lori A. Zoellner, Jacob A. Bentley, Kawther Musa, Farhiya Mohamed, Luul B. Ahmed, Kevin M. King, Norah C. Feeny

**Affiliations:** 1 Department of Psychology, University of Washington, Seattle; 2 Department of Rehabilitation Medicine, School of Medicine, University of Washington, Seattle; 3 Somali Community, Columbus, Ohio; 4 Somali Family Safety Task Force, Seattle, Washington; 5 Our Health of Ohioans, Columbus, Ohio; 6 Department of Psychological Sciences, Case Western Reserve University, Cleveland, Ohio

## Abstract

**Importance:**

The global refugee crisis disproportionately affects the Muslim world. Forced displacement often results in trauma-related mental health issues. Effective psychotherapy exists, but there are barriers to uptake by refugee groups as well as a lack of culturally appropriate interventions.

**Objective:**

To examine the efficacy of a brief, lay-led, mosque-based intervention, Islamic Trauma Healing (ITH), adapting empirically supported cognitive behavioral principles to improve mental health and well-being.

**Design, Setting, and Participants:**

In a randomized clinical trial, 101 participants received ITH or active assessment but delayed intervention (waiting list [WL]) in mosques and virtually in Seattle, Washington, and Columbus, Ohio, and were assessed through 12-week follow-up. Data were collected from July 14, 2018, through July 14, 2022, and data analysis was conducted from March 13 to July 31, 2023. United States–based refugees from Somalia who experienced a *Diagnostic and Statistical Manual of Mental Disorders, Fifth Edition* (*DSM-5*) Criterion A trauma exposure and posttraumatic stress disorder (PTSD) reexperiencing or avoidance. Analyses were intention-to-treat, using full information likelihood for missing data.

**Intervention:**

Islamic Trauma Healing included psychoeducation, discussion of the lives of prophets who had undergone trauma, and informal prayer turning to Allah about the trauma, incorporating cognitive restructuring and imaginal exposure. Lay-leader training is purposely brief: two 4-hour sessions with weekly supervision.

**Main Outcomes and Measures:**

The primary outcome was PTSD severity (measured with the Posttraumatic Diagnostic Scale for *DSM-5* [PDS-5]). Secondary outcomes included depression (Patient Health Questionnaire-9), somatic symptoms (Somatic Symptoms Severity-8), and quality of well-being (World Health Organization Five Well-Being Index). Analyses were intention-to-treat.

**Results:**

Analyses were based on all 101 randomized participants (92 [91.1%] women; 9 [8.9%] men; mean [SD] age, 46.5 [12.02] years) with baseline mean (SD) PDS-5 score of 31.62 (16.55) points. There were significant differences in PTSD severity (*d* = −0.67), depression (*d* = −0.66), and well-being (*d* = 0.71), comparing ITH vs WL after the intervention. Gains were maintained through 12-week follow-up. Islamic Trauma Healing was consistent with religious and cultural practices (mean [SD], 3.8 [0.43]) and promoted community reconciliation (mean [SD], 3.8 [0.42]).

**Conclusions and Relevance:**

In this randomized clinical trial of a brief, lay-led mental health intervention, ITH proved superior to WL. The findings suggest that ITH has the potential to provide an easily trainable and scalable intervention, incorporating Islam and empirically supported principles, that addresses the psychological wounds of war and refugee trauma.

**Trial Registration:**

ClinicalTrials.gov Identifier: NCT03502278

## Key Points

**Question:**

Does a brief, lay-led intervention for war and refugee trauma incorporating cognitive behavioral and Islamic principles have efficacy to address trauma-related mental health issues and well-being, compared with a control group?

**Findings:**

In a randomized clinical trial with a US-based sample of 101 refugees, Islamic Trauma Healing showed significant effects on posttraumatic stress disorder, depression, and well-being, compared with the control group, with gains maintained through 12-week follow-up. Islamic Trauma Healing was well received by community members, consistent with faith and culture.

**Meaning:**

The findings of this study suggest that this deep-cultural–adapted intervention has the potential to provide an easily trainable and scalable model to reach war and refugee communities unlikely to either have access to or seek Western-based mental health interventions.

## Teaser

This randomized clinical trial evaluates the use of small-group intervention led by lay persons for psychological trauma experienced by Islamic refugees, maintaining religious and cultural standards.

## Introduction

The United Nations High Commissioner for Refugees estimates that more than 103 million individuals are forcibly displaced worldwide,^1^ many of whom come from Muslim majority or war-torn countries. Individuals who have been forcibly displaced often report high levels of trauma exposure and trauma-related mental health issues, including posttraumatic stress disorder (PTSD) and depression.^2,3^ Furthermore, multiple trauma exposures, including during migration, places displaced persons at even higher risk for mental health problems.^4,5^

Substantial barriers to mental health care exist for displaced persons. These include resource and mental health access limitations,^6,7,8^ language and trust barriers,^9^ explanatory models that differ from predominant Western biomedical perspectives or perceived misalignment with one’s faith,^10,11,12,13^ lack of culturally sensitive interventions and treatment professionals,^14^ and potential stigma of mental health symptoms and treatment-seeking.^15,16,17^

Empirically supported psychotherapies for trauma-related mental health issues exist.^18,19^ Of these psychotherapies, prolonged exposure and cognitive processing therapy emerge across evidentiary metrics as strong interventions, outperforming other PTSD psychotherapies^20^ and demonstrating long-term maintenance of gains.^21^ Key mechanisms of these interventions point to the role of approaching trauma-related memories and reminders and challenging negative trauma-related beliefs.^22,23,24^ However, these interventions are typically conducted individually, rely upon professionally trained and credentialed clinicians, and require extensive supervision, impacting reach and scalability to populations and settings with resource constraints.

None of these interventions has an Islamic focus, despite more than 25% (>1.7 billion) of the world’s population practicing Islam.^25^ In many Muslim communities, mosques are the center of community life and provide existing infrastructures to easily offer and scale-up services. Islamic adaptations of cognitive-behavioral psychotherapy have been developed for anxiety, depression, and traumatic stress,^26,27^ with, to our knowledge, no randomized clinical trials on PTSD to date. These adaptations were designed for use by credentialed professionals. Peer-delivered interventions have the potential to address language and cultural barriers as well as a lack of available professionals.^28^ In a nonrandomized study of a peer-delivered cognitive-behavioral psychotherapy for Somali women in Minnesota (N = 55), mood and anxiety improved,^29^ but perceived inconsistency with Islam was noted as a major barrier to uptake.

Islamic Trauma Healing (ITH) is a lay-led, small-group intervention specifically targeting healing mental wounds of trauma. It was initially developed within the Somali community^30^ to address mental health–seeking barriers.^11,12,15^ The 6-session intervention combines empirically supported exposure-based and cognitive restructuring techniques derived from first-line cognitive behavioral psychotherapy for PTSD (ie, prolonged exposure and cognitive processing therapy), with Islamic principles. Acknowledging the effect of trauma on the community, ITH further promotes community building, reconciliation, and posttraumatic growth. Training time is purposely brief: two 4-hour sessions. Preliminary quantitative and qualitative trial data from the US and Somaliland showed a strong perceived need and match with the Islamic faith, large reductions from before to after intervention across symptom measures, and ease of remote supervision.^31,32,33^ The present study is, to our knowledge, the first randomized clinical trial of ITH for trauma-related mental health issues, comparing ITH with a waiting list control condition (WL) in a sample of Somali refugees in the US. It was hypothesized that ITH would be superior to WL across PTSD, depression, somatic symptoms, and well-being outcomes and that these gains would be maintained through follow-up.

## Methods

This randomized clinical trial was approved by the University of Washington and Case Western Reserve University institutional review boards. The trial protocol is available in Supplement 1. All participants gave written informed consent. Participants received $50 paid at follow-up. This study is reported following the Consolidated Standards of Reporting Trials (CONSORT) reporting guideline.

### Participants

Men and women with trauma exposure and trauma-related avoidance or reexperience participated in the study. Inclusion and exclusion criteria were carefully selected to be easily behaviorally recognized to help individuals self-identify and to not include structured diagnostic interviews to reduce potential stigma. If the groups become known by the community as only for patients with psychiatric disorders, many individuals in need of help might not participate. Inclusion criteria included *Diagnostic and Statistical Manual of Mental Disorders, Fifth Edition* (*DSM-5*) definition of Criterion A trauma exposure at least 12 weeks earlier, either avoidance and reexperiencing PTSD symptoms, being of the Islamic faith, and between the ages of 18 and 65 years. Exclusion criteria included observed cognitive impairment, inability to participate in group discussions, or current suicidal intent or plan.

### Measures

Consistent with a transdiagnostic approach,^34^ chronic psychopathologic factors following trauma were assessed as dimensional and multifaceted. The primary outcome was PTSD severity (Posttraumatic Diagnostic Scale for *DSM-5* [PDS-5]) (score range, 0-80; higher scores indicate greater severity).^35^ Secondary outcomes included depression (Patient Health Questionnaire-9) (score range, 0-27; higher scores indicate greater severity),^36^ somatic symptoms (Somatic Symptoms Severity-8 [SSS-8]) (score range, 0-16; higher scores indicate greater severity),^37^ and quality of well-being (World Health Organization Five Well-Being Index [WHO-5]; score range 0-25 [higher scores indicate better functioning]).^38^ Satisfaction was assessed with items derived from the Client Services Satisfaction Questionnaire.^39^ Measures were translated and back-translated from English to Somali, with Somali audio versions.

### Intervention

#### Islamic Trauma Healing

Islamic Trauma Healing is a 6-session, lay-led group, manual-based intervention.^32,33^ Small groups were conducted in mosques and virtually with approximately 5 to 7 same-gender members, with 2 lay leaders of the same gender. Islamic Trauma Healing combines techniques from evidence-based trauma-focused, cognitive behavioral psychotherapy, targeting unhelpful negative beliefs and trauma-related avoidance with Islamic principles and practices. Sessions, conducted in Somali, included time for community building rituals (eg, sharing tea and snacks), spiritual preparation using a brief supplication, and a brief closing supplication. The Islamic content was carefully vetted and focused on central tenets to be applicable across Islamic schools and branches.^30^ At each session, prophet narratives were read out loud, including Qur’an verses of prophets’ lives who had experienced trauma (eg, Prophets Ayyub [Job] and Yusuf [Joseph]). Participants also spent time in individual, informal prayer, turning to Allah in dua about their trauma, with instructions encouraging the approach of the trauma memory (ie, imaginal exposure). Cognitive restructuring–focused discussion questions followed both the prophet narratives and dua, guiding perspective-taking and meaning-making of the trauma. Content shifted from the presence and purpose of suffering to healing and reconciliation for oneself, others, and the larger community.

##### Leader Training, Fidelity, and Clinical Supervision

Lay leaders were selected for a heart for healing, knowledge of the Qur’an, being respected in their community, and reasonably fluent in Somali and English, but no formal mental health training. They were trained in two 4-hour sessions by PhD-level clinical psychologists (L.A.Z., J.A.B., and N.C.F.), focusing on discussion-leading skills and familiarity with components; the manual provided verbatim wording to be read to the group for key therapeutic elements. After each session, leaders met with a clinical supervisor, discussed the session, and completed a detailed session-specific fidelity checklist, with a licensed clinical psychologist (L.A.Z., J.A.B., and N.C.F.) providing clinical oversight. Fidelity was excellent, with 94.1% of essential elements (common reactions to trauma, prophet narratives, and turning to Allah in dua) completed at an adequate time duration. No protocol deviations were observed and no changes to the intervention were made from the protocol.

#### Waiting List, Active Assessment Control Condition

Waiting list groups were repeatedly assessed at weeks 0, 3, and 6, coming together as a group with their eventual leaders with water, tea, and/or snacks. After week 6, WL groups received ITH, with their final post-ITH assessment at week 12 follow-up.

### Procedures

Data were collected from July 14, 2018, through July 14, 2022. Participants were recruited through flyers and word of mouth by community and faith leaders and self-referral. Recruitment for the trial continued until all recruitment goals and follow-up assessments were achieved. Informed consent was completed in either Somali or English and explained and read out loud, if needed. Measures were completed via paper or electronic device. If the PHQ suicide item was indicated, further screening occurred. Randomization to the active intervention or WL was conducted using Medsharing Randomizer for Clinical Trial Lite by Regis Bournique application,^40^ with a 1:1 ratio, held by the study coordinator blinded to condition until the time of randomization, allowing for individuals or clusters of up to 3 individuals being randomized as a unit. Randomization was concealed until participants were enrolled and assigned to conditions. After randomization, ITH groups, conducted in Somali, met weekly for approximately 2 hours, with length varying based on daily prayer times. After each group, lay leaders met briefly with a clinical supervisor. After session 6, a group social event was held as a closure ceremony and to facilitate community reconciliation. Measures were completed at baseline, week 3, week 6, and a 12-week follow-up. After week 6, individuals in the WL received ITH, with their post ITH assessment being the 12-week follow-up.

### Statistical Analysis

Data analysis was conducted from March 13 to July 31, 2023. Power was estimated to detect effects using G*Power and Optimal Design (for multilevel data), with α = .05 and power (1 – β) = 0.80, completing 10 000 replications per analysis. With an estimated dropout level of 20%, we had power to detect moderate to large effects of condition with 30 individuals per group (*f^2^* = 0.28).

Analyses were intention-to-treat, using full information likelihood for missing data. R, version 4.2.2. (R Foundation for Statistical Computing) was used for the main analyses,^41^ using nlme^42^ and psych.^43^ To test the main hypotheses, general linear mixed models were used, accounting for clustering within individual groups and controlling for the community (Seattle and Columbus), and accounting for change from baseline to posttreatment. Time was coded so the intercept reflected the level of the outcome at postintervention (eg, −2 = baseline, −1 = mid-intervention, 0 = postintervention), allowing the effect of the intervention to be interpreted as differences in groups at postintervention, while a time × condition interaction obtained estimates of baseline to postchange for each condition. Ten participants chose to be randomized based on cluster; thus, clustering within this unit was not feasible. The magnitude of pretest to posttest change within both conditions was estimated and the stability of symptoms from posttest to follow-up was examined. In addition, general linear mixed models were used to estimate posttest to follow-up change for the WL, once ITH was implemented within that condition, with the intercept set to the follow-up time point, and again estimating a time by condition interaction to obtain the differences in change from posttest to follow-up across conditions.

## Results

**Figure 1.**
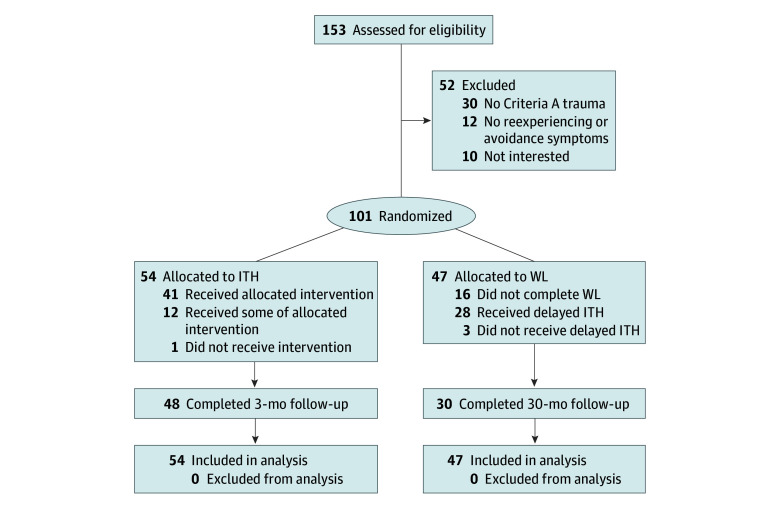
Consolidated Standards of Reporting Trials Diagram ITH indicates Islamic Trauma Healing; WL, waiting list.

Analysis was based on all 101 randomized participants (92 [91.1%] women and 9 [8.9%] men) (Table 1). The mean (SD) age of the sample was 46.5 (12.02) years (range, 20-86 years). Nearly all individuals in the sample were Black (99 [98.0%]) and originally from Somalia (100 [99.0%]). The participants spent a mean (SD) of 6.61 (5.35) years in migration (range, 1-24 years) and 10.81 (6.72) years in the US (range, 1-27 years). Most participants (56 [55.4%]) reported their index *DSM-5* Criterion A trauma as being exposed to a war zone or military combat. The PTSD severity level at baseline was in the moderate clinical range, with a mean (SD) PDS-5 score of 31.62 (16.55) points. Fifty-four participants were randomized to immediate ITH and 47 to the WL. Of those in the WL, after postintervention, 28 received delayed ITH (Figure 1).

**Table 1.** Demographic Characteristics

Characteristic	Participants, No. (%)	
	ITH (n = 54)	WL (n = 47)^a^
Age, mean (SD), y^b^	47.4 (13.1)	45.4 (10.7)
Gender		
Female	48 (88.9)	44 (93.7)
Male	6 (11.1)	3 (6.3)
Country of origin (Somalia)	54 (100)	46 (97.9)
Race (Black, African, African American)^c^	52 (96.3)	47 (100)
Relationship status (married)	29 (53.7)	21 (45.7)
Educational level (high school diploma or higher)	7 (13.3)	9 (17.3)
Years in migration to US, mean (SD) y	6.4 (5.3)	6.8 (5.5)
Years in US, mean (SD) y	10.6 (6.3)	11.1 (7.2)
Index *DSM-5* criterion a trauma exposure		
Serious life-threatening illness	4 (7.4)	2 (4.3)
Physical assault	3 (5.6)	7 (14.9)
Sexual assault	3 (5.6)	4 (8.5)
War zone or military combat	34 (63.0)	22 (46.8)
Child abuse	2 (3.7)	2 (4.3)
Accident	5 (9.3)	3 (6.4)
Natural disaster	3 (5.6)	4 (8.5)
Other trauma	0	3 (6.4)

Abbreviations: *DSM-5*, *Diagnostic and Statistical Manual of Mental Disorders, Fifth Edition*; ITH, Islamic Therapeutic Healing; WL, waiting list.

^a^ Repeated assessment control group.

^b^ Records of dates of birth often do not exist and individuals could only approximate age.

^c^ Self-reported.

### Effects of ITH vs WL at Postintervention

At postintervention (week 6), there were significant and substantial differences between ITH and WL on all outcomes except somatic symptoms. At postintervention, participants in the ITH reported lower levels of posttraumatic stress symptoms than those in the WL (PDS-5: *d* = −0.67; estimate = −11.12; SE = 3.83; *P* = .01), depressive symptoms (PHQ-9: *d* = −0.66; estimate = −4.0; SE = 1.41; *P* = .01), and higher levels of well-being (WHO-5: *d* = 0.71; estimate = 4.00; SE = 1.56; *P* = .02). There were no significant differences in somatic symptoms between the ITH and WL groups after the intervention (SSS-8: *d* = −0.22; estimate = −0.84; SE = 1.42; *P* = .56).

**Figure 2.**
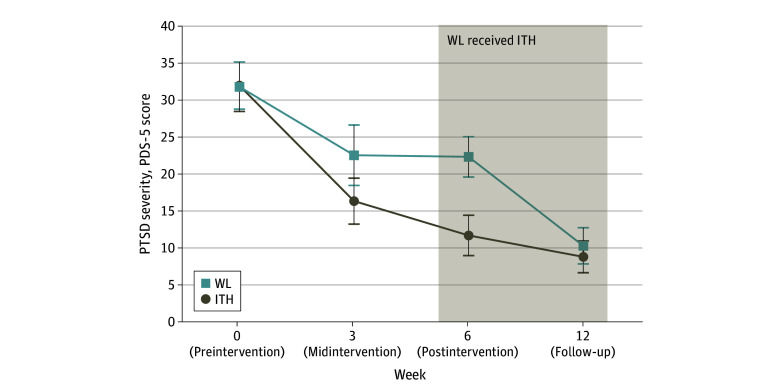
Islamic Trauma Healing (ITH) vs Waiting List (WL) in Primary Outcome Posttraumatic Stress Disorder (PTSD) Severity PDS-5 indicates Posttraumatic Diagnostic Scale for *Diagnostic and Statistical Manual of Mental Disorders, Fifth Edition*.

Table 2 provides estimated means (SEs) over time. For active ITH, there were large within-group changes from before to after the intervention for PTSD severity (PDS-5: *d* = −1.80), depression severity (PHQ-9: *d* = −0.97), well-being (WHO-5: *d* = 1.11), and moderate before to after effects for somatic symptoms (SSS-8: *d* = −0.49) (Figure 2).

**Table 2.** Primary and Secondary Outcomes Comparing ITH vs WL Through Follow-Up Intention-to-Treat Analysis^a^

Measure	Week, mean (SE)			
	0 (Baseline)	3	6 (Postintervention)^b^	12 (Follow-up)
**Islamic Trauma Healing**				
Primary outcome				
PTSD severity (PDS-5)^c^	31.99 (3.19)	16.35 (3.11)	11.71 (2.73)	8.81 (2.15)
Secondary outcome				
Depression (PHQ-9)^d^	8.21 (1.08)	5.32 (1.02)	3.54 (0.90)	4.00 (0.89)
Somatic symptoms (SSS-8)^e^	5.89 (0.72)	4.36 (1.11)	4.18 (0.84)	3.61 (0.59)
Well-being (WHO-5)^f^	16.49 (0.90)	19.33 (1.12)	21.32 (0.99)	20.10 (0.84)
**Waiting list**				
Primary outcome				
PTSD severity (PDS-5)^c^	31.83 (3.34)	22.56 (4.09)	22.34 (2.73)	10.30 (2.45)
Secondary outcome				
Depression (PHQ-9)^d^	8.01 (1.14)	8.33 (1.35)	7.30 (1.04)	3.19 (1.02)
Somatic symptoms (SSS-8)^e^	5.20 (0.76)	4.16 (1.39)	5.12 (0.98)	2.30 (0.70)
Well-being (WHO-5)^f^	18.74 (0.95)	18.98 (1.48)	17.16 (1.17)	22.18 (1.01)

Abbreviations: *DSM-5*, *Diagnostic and Statistical Manual of Mental Disorders, Fifth Edition*; PDS-5, Posttraumatic Diagnostic Scale for *DSM-5*; PHQ-9, Patient Health Questionnaire-9; PTSD, posttraumatic stress disorder; SSS-8, Somatic Symptoms Severity-8; WHO-5, World Health Organization Five Well-Being Index.

^a^ Data estimated from the multilevel models (N = 101).

^b^ At 6 weeks, WL shifted to receiving active ITH.

^c^ PDS-5, score range, 0 to 80; higher scores indicate greater severity.

^d^ PHQ-9, score range, 0 to 2; higher scores indicate greater severity.

^e^ SSS-8, score range, 0 to 16; higher scores indicate greater severity.

^f^ WHO-5, score range, 0 to 25; higher scores indicate better functioning.

### Dropout and Clinically Meaningful Gains

There was no significant difference between those who completed active ITH (≥4 sessions, 76.0% [n = 41]) compared with WL (66.0% [n = 31]) (χ^2^ [1, n = 101] = 1.22; *P* = .27). There were no adverse or severe adverse events. After the intervention (week 6), 14.8% of individuals in the ITH group met cutoff criteria for PTSD diagnosis^35^ compared with 40.4% of the WL group (χ^2^ [1, n = 10] = 8.42; *P* = .004; number needed to treat = 3.9).

For those in active ITH who completed the intervention, exploratory analyses showed higher baseline severity was associated with a larger change from preintervention to postintervention (PDS-5: *r* = 0.81; *P* < .001; PHQ-9: *r* = 0.83; *P* < .001; SSS-8: *r* = 0.61; *P* < .001; WHO-5: *r* = 0.80; *P* < .001). These findings suggest that the greater benefit was for those with more severe symptoms.

### Maintenance of ITH Gains Through Follow-Up

During follow-up, those in the WL group received ITH and showed moderate to large gains from postintervention to follow-up (PDS-5: *d* = −0.64; estimate = −10.57; SE = 2.05; *P* < .001; PHQ-9: *d* = −0.64; estimate = −3.93; SE = 0.83; *P* < .001; WHO-5: *d* = 0.84; estimate = 4.88; SE = 1.15; *P* < .001; SSS-8: *d* = −0.73; estimate = −2.84; SE = 0.72; *P* < .001). Furthermore, at follow-up, there were no significant differences between ITH and WL, with WL members receiving ITH (*d* = −0.32 to 0.04; *P* = .17-.86). These findings suggest that individuals initially receiving ITH maintained their gains through follow-up.

### ITH Program Satisfaction

On a scale of 1 (poor) to 4 (excellent), participants reported that ITH matched well with their religious beliefs and cultural practices (mean [SD], 3.8 [0.43]), helped with trauma-related healing (mean [SD], 3.8 [0.45]), will help with community reconciliation (mean [SD], 3.8 [0.42]), would recommend to a friend in need (mean [SD], 3.6 [0.79]), and were satisfied overall (mean [SD], 3.7 [0.71).

## Discussion

In a sample of people who were displaced due to war and refugee trauma, this randomized clinical trial of a brief, lay-led intervention showed moderate to large effects of ITH compared with a repeated assessment WL condition for PTSD, depression, and well-being, with gains maintained during follow-up. Large, clinically meaningful, preintervention to postintervention improvements were observed for ITH, with effect sizes benchmarking well with trials using well-trained professionals, and 12 to 20 individual psychotherapy sessions, using first-line interventions.^44^

The international community faces a dilemma in addressing the mental health needs of individuals from humanitarian crisis–affected low- and middle-income countries and resultant refugee populations.^45,46,47^ While other brief, effective Sub-Saharan Africa–focused and refugee-focused interventions exist,^48,49^ ITH is unique in that it is embedded within faith communities to facilitate trauma healing and community reconciliation, shifting the focus from mental health professionals to communities and mosques, and to promote community reconciliation. Islamic Trauma Healing also directly addresses stigma, normalizing the influence of trauma on the community. The program does not train community members to be psychotherapists or paraprofessionals, but trains discussion leaders, with a brief training of two 4-hour sessions, which is a considerable reduction from other interventions.^48,49^ These ITH features may facilitate community uptake and scalability.

Islamic Trauma Healing was well liked, having a strong match with religious beliefs and cultural practices. Qualitative feedback mirrored satisfaction ratings, suggesting “I like every part. . . Please don’t stop this program, our people need this program.” Key components that were most helpful included “. . .the religious focus part of the trauma, especially the stories of the prophet,” and “our social connection…we were strangers and now…we’re together.” These statements map well with potential therapeutic mechanisms highlighting the role of religious faith and spiritual practices,^50^ shifts in trauma-related beliefs about oneself and the world,^24^ and enhancing interconnectedness and social support.^51^

### Strengths and Limitations

The current trial is, to our knowledge, the first randomized clinical trial of ITH, including a repeated assessment the WL and a brief follow-up period. Future trials will need to use an active comparator, evaluation of implementation-oriented outcomes, longer-term follow-up, and cost-effectiveness. The WL was assessed repeatedly as a group and eventually received ITH, helping control for social connection and expectancy. Conditions did not differ significantly in dropout, with numbers in the opposite direction showing higher rates for the WL than the trauma-focused intervention. Participants were not selected for a PTSD diagnosis, and well-trained, blinded interviewers were not employed. This was a strategic decision; working with the community to not stigmatize members or have the groups labeled as having psychiatric disorders. Furthermore, there is strong evidence for correspondence across PTSD self-report and interview measures.^35,52^

Only moderate effects were observed for somatic symptoms, and no major differences between ITH and WL were observed. Despite elevations of somatic symptoms in refugee samples,^53^ in the present study, the modal baseline response was “not bothered” and mirrored normative, not psychosomatic, samples.^37,54^ Thus, the lack of initial elevation likely provided no room to detect change. Within this refugee community, having to wait even 6 weeks was not viewed as acceptable, contributing to the lack of WL retention. Recruitment of men was low, unlike in a previous trial,^33^ and limits generalizability. This may reflect higher perceived need in women (and higher rates of PTSD and depression), predominantly female lay leaders, and competing financial demands. In addition, while the intervention, with appropriate cultural adaptation, was developed for potential use across Muslim trauma-exposed populations, the results may not generalize to other Muslim communities or refugee communities that are not Muslim.

## Conclusions

In this randomized clinical trial, this novel ITH intervention empowers Muslim communities to address the individual and collective impact of war and refugee trauma. Islamic Trauma Healing integrates the role of faith in shaping how trauma shifts views of oneself, the world, and other people by incorporating Islamic principles with empirically supported psychotherapy practices to address the complex mental health needs of people who are displaced. In many respects, refugee communities and their leaders are in the best position to build trust and relationships necessary for mental health help-seeking. Evidence from 2 pilot trials of ITH^32,33^ and this randomized clinical trial show large, clinically meaningful gains in the hands of briefly trained lay persons both in the US and Somalia. Replication and cultural adaptation with other samples are needed, as well as for other forms of humanitarian crisis. Islamic Trauma Healing has the potential to serve as a standalone intervention or part of a stepped-care model for entry into individual psychotherapy, being able to be completed in 1, 2, 3, or 6 weeks. Key innovations include a deep cultural, faith-based adaptation of evidenced-based psychotherapy for war and refugee trauma, integration within existing community infrastructure, brief lay person training time, low supervision burden, and potential increased ease and reduced cost to scale for humanitarian crisis settings and refugee camps where access to mental health professionals is limited.

## Notes

Supplement 1.
Trial Protocol

Supplement 2. **Nonauthor Collaborators**. The Islamic Trauma Healing Clinical Team

Supplement 3.
Data Sharing Statement
