# Error in Figure 2

**DOI:** 10.1001/jamanetworkopen.2024.40198

**Published:** 2024-09-24

In the Original Investigation titled “Lay-Led Intervention for War and Refugee Trauma: A Randomized Clinical Trial,”^1^ published August 26, 2024, there was a labelling error in Figure 2. In the key, the color labelled “ITH” should have been labelled “WL,” and the color labelled “WL” should have been labelled “ITH.” This article was corrected.^1^
