# Supplementary material for: Lay-Led Intervention for War and Refugee Trauma: A Randomized Clinical Trial
Source: JAMA Netw Open. 2024 Aug 26;7(8):e2429661. doi: 10.1001/jamanetworkopen.2024.29661 (PMC11423170; doi:10.1001/jamanetworkopen.2024.29661)
Supplement: Supplement 1. — Trial Protocol [file jamanetwopen-e2429661-s001.pdf]

STUDY PROTOCOL: A Lay-Led Intervention for War and Refugee Related Trauma: NCT03502278
July 18, 2018

A. Participants: U.S. Somali Refugee Community Sample

We will conduct this study in Seattle, WA, and Columbus, OH. The study will be a small RCT (*N* = 60) comparing Islamic Trauma Healing to waitlist (WL) (1:1 randomization, using cluster randomization of one to three individuals), with a three-month follow-up.

Inclusion and Exclusion Criteria. Men and women with trauma exposure and trauma-related avoidance or reexperiencing will participate in the study. Inclusion and exclusion criteria were carefully selected to help individuals self-identify as needing the program while also not conducting formal diagnostic measures that could stigmatize or alienate potential group members. Specifically, if the groups become known as groups for "patients" or for the "insane," many individuals in need of help will not participate. Concrete, behaviorally obvious inclusion criteria where chosen to facilitate lay leader and self-referral. DSM-5 definition of trauma exposure allows for a variety of events including witnessing or experiencing "life threatening events." Two behavioral symptoms of PTSD were chosen, specifically avoidance and re-experiencing symptoms, that are common, readily recognized, often easily linked to trauma exposure; and accordingly, easy for individuals to identify.

Table 2. Broad and Generalizable Criteria
Inclusion:
- Experienced a DSM-5 trauma at least 12 weeks ago
- Report current re-experiencing or avoidance symptoms
- Islamic faith
- 18-65 year of age

Exclusion:
- Immediate suicide risk, with intent or plan
- Cannot understand consent/visible cognitive impairment

Potential participants will self-identify as needing trauma healing. We chose not to use formal PTSD and depression interview diagnostic measures for several reasons. Somali refugees may not identify or report many of the symptoms associated with a Western conceptualization of PTSD and depression (Bentley et al., 2011). Further, in line with dimensional models of psychopathology, subthreshold PTSD symptoms are also common, often unremitting, and impairing and also warrant intervention (Bergman et al., 2016). By not requiring a DSM-5 diagnosis of PTSD, a broader conceptualization of post-trauma reactions and associated phenotypes is in line with RDoC Negative Valence Systems and a better match with current thought on the wide range of long-term psychopathology following trauma exposure (Kozak & Cuthbert, 2016, Broman-Fulks et al., 2006).

Participants will be of the Islamic faith, as the intervention will be conducted in mosques and uses tenets of the Islamic faith to promote trauma healing. Participants will be expected to not be visibly cognitively impaired. Individuals with current suicidal intent or plan will be excluded and provided appropriate referrals. Referral sources are well known in Seattle and Columbus. See Table 2 for Inclusion/Exclusion criteria.

B. Materials

***Islahul Qulub*: Islamic Trauma Healing.** Several years of iterative, collaborative development work with the Somali community have guided the content and format of the Islamic Trauma Healing program. A manual for the program was developed (Lang, Zoellner, Graham, Marks, & Feeny, 2016), and a local Imam carefully reviewed the content of the manual. Focus group feedback from lay leaders and group members was also utilized in revisions. The program was designed for groups of 5-7 members, with two lay leaders of the same gender. The program is structured so that the lay leader training, comprised of two 4-hour trainings, focuses on teaching lay leaders the skills of discussion leading, with the manual content providing more of the direct therapeutic work. That is, the lay leaders are not explicitly taught how to be psychotherapists or how to do cognitive behavioral therapy specifically. The manual contains an introduction to trauma healing, including a description of types of trauma exposure and common reactions, as well as Islamic principles related to trauma healing. Session-by-session content is clearly spelled out in the manual. Each session includes time for community building rituals (e.g., sharing tea or coffee), spiritual preparation using a brief supplication written by the local Imam, prophet narratives relevant to trauma healing, and a brief closing supplication also written by the Imam. In the first session, a rationale for the program, common reactions to trauma, and a breathing relaxation exercise are described. Starting in the second session and continuing through the last session, talking individually to Allah about the trauma is conducted. In the last session, group members are encouraged to organize a closing event at which certificates of program completion are given.

The program is 6 sessions long. This duration is in line with evidence that very brief or shortened protocols (e.g., four 30 min sessions) can substantially reduce PTSD symptoms (Cicrang et al., 2011; van Minnen & Foa, 2006). Key components of the program are prophet narratives aimed at targeting trauma-

related beliefs, and talking to Allah, aimed at targeting trauma memories.

Prophet Narratives and Group Discussion. Prophet narratives are brief synopses of a particular prophet's life, including Qur'an verses. Cognitive restructuring-related group questions follow the narrative, mirroring the theme for the session. Prophet narrative content and questions shift from the presence and purpose of suffering to healing and reconciliation for oneself, others, and the larger community. These include: Session 1, Faith During Hard Times Prophet Job (Ayyub); Session 2, Trials Build Strength Prophet Joseph (Yusuf); Session 3, Overcoming Fear Prophet Moses (Muses); Session 4, Redemption of Self and Others Prophet Jonah (Yoonus); Session 5, Faith, Courage, and Hope for the Future Prophet Abraham (Ibraheem); and Session 6, Reconciliation Prophet Muhammad [peace be upon him]. Prophet narratives are read aloud (5-10 mins) by the group leaders. Following each narrative are questions to facilitate a group discussion related to the theme. The themes form an arch, moving from suffering to healing to growth following trauma. Many people of the Islamic faith are accustomed to talking about prophet stories, making this well aligned with their faith and would not be considered 'unusual' by group members or others hearing about the group.

Talking to Allah and Group Discussion. From sessions 2-6, participants are asked to spend time in individual prayer, talking to Allah about their trauma. Of note, the term 'prayer' is used illustratively, having varied meanings within Islamic practice, with the manual specifically using the term "talking to Allah." This prayer time is conceptualized as an adapted form of imaginal exposure to the trauma memory(ies). Muslims have a call to pray five times per day, and in our pilot work, talking with Allah about personal experiences, including the experience of trauma, is very intuitive. In the first session, group leaders provide a rationale for talking to Allah. In the second session, this rationale is repeated, including instructions about how to select a trauma memory, and an example prayer is provided. Talking to Allah is conducted individually for approximately 15-20 min. Content of the prayer shifts from simply talking about what happened, talking to Allah about feelings and thoughts experienced during the trauma, to talking to Allah about the hardest parts, to finally thanking and praising Allah for the experience of what he or she has learned through the trauma and through talking about it. Following individual prayer are questions to facilitate a group discussion related to the theme. Participants are encouraged to talk in the group about their experience while talking to Allah but not to directly share their traumatic experiences with the group during this time. This is intended to promote cognitive restructuring of negative trauma-related beliefs and foster social connectedness among group members. Similarly, the content of the talking to Allah forms an arch from initially approaching the trauma memory to approaching the hardest parts of the memory to at the end shifting the meaning of the memory to have positive or growth elements to it.

C. Measures

The following questionnaires have all been translated and back-translated from English to Somali, with audio versions of the questions in Somali. Questionnaires will be completed electronically using a mobile device (e.g., tablet, smartphone) and headphones with Qualtrics Programming. Diagnostic interview measures are not included, due to cultural stigma associated with mental healthcare, including getting any kind of formal diagnostic assessment (Aloud & Rathur, 2009). We want to avoid community members associating the group with PTSD, as we believe that will decrease interest in participating due to well-known cultural stigma against mental healthcare (e.g., Aloud & Rathur, 2009). Diagnostic measures also have issues with sustainability; requiring additional training of lay assessors who will conduct clinical interviews once the program is self-sufficient. Furthermore, we want to ensure that the intervention works for individuals with a range of symptom levels from mild to severe, but that at a minimum hallmark symptoms of PTSD are reported (i.e., avoidance or reexperiencing).

Measures were selected to be minimal and efficient to target key constructs of interest. Low literacy in this sample is a considerable issue, both in English and in Somali, resulting in longer measure completion times. It should be noted that we originally used paper and pencil measures in both English and Somali, resulting in items needing to be read and long completion times. When we shifted to electronic questionnaires in both English and Somali together, with audio files in Somali for each question, completion time decreased substantially and participant satisfaction also increased.

Main Outcome Measures. Consistent with an RDoC approach, chronic psychopathology following trauma is assessed as dimensional and multifaceted, examining not only PTSD, but also depression, somatic symptoms, and functional wellbeing. Although multiple units of analysis were considered (e.g., cortisol, blood pressure, genetics), considering the cost/benefit ratio, their addition was not strongly scientifically justified.

PTSD Scale – Self-Report for DSM-5 (PS-SR-5; Foa et al., 2015). This measure provides a trauma screen and rates DSM-5 symptoms for the last 2 weeks, including functional impairment. This measure has good reliability and inter-rater reliability.

Patient Health Questionnaire-9 for depression symptoms (PHQ-9; Kroenke, Spitzer, & Williams, 2001). The PHQ-9 is a self-report measure of depression symptoms with each question rated from 0-3. This measure has shown very good internal consistency (α = .89), and demonstrates good discriminative validity (area under curve = 0.95) and correlates moderately to strongly with the SF-20 (Kroenke, Spitzer, & Williams, 2001). This measure will also be used for monitoring suicidal thoughts. The addition of a measure specific to suicide has the potential to be off-putting or even stigmatizing for the program given cultural beliefs on the topic and substantially increases participant burden.

Patient Health Questionnaire-15 for somatic symptoms (PHQ-15; Kroenke, Spitzer, & Williams, 2002). The PHQ-15 is a self-report assessment of somatic symptoms (e.g., stomach pain, headaches, dizziness) in the past four weeks. Symptoms are rated as "*not bothered at all*," "*bothered a little*," or "*bothered a lot*." This measure will use an abbreviated 8-item version and is included as a main outcome, given the likelihood of somatization of trauma-related symptoms in this population (Bentley, Thoburn, Stewart, & Boynton, 2011). The measure shows good reliability (α = .80) and construct validity given associations with mean disability days, symptom-related difficulty, and mean physician visits.

WHO-5 Wellbeing Index (WHO-5; Bech, Olsen, Kjoller, & Rasmussen, 2003). This five-item measure assesses emotional well-being on a 0-5 scale over the past two weeks. It shows good convergence with the PHQ-9 and SF-12 (r = .55 - .69) and has good sensitivity and specificity (Hajos et al., 2013).

Client Services Satisfaction Questionnaire (Larsen, Attkisson, Hargreaves, & Nguyen, 1979). This measure is included to assess acceptability of content and delivery of this novel program. The questionnaire was modified to contain 5 items, scored from 1 (*poor*) to 4 (*excellent*). The original CSQ, an 8-item self-report measure of satisfaction with health and human services, showed very good internal consistency (α = .92-.93) and appeared to have sound construct validity, as evidenced by relationships with lower CSQ score/higher dropout, and lower CSQ score/more appointments missed.

Mechanism Measures. There is very little work in refugee or Somalian samples. We will conduct psychometric analyses of each measure to guide needed adaptations for the subsequent R01.

Posttraumatic Cognitions Inventory (PTCI; Foa, Ehlers, Clark, Tolin, & Orsillo, 1999). This self-report measure assesses maladaptive trauma-related beliefs about self, the world, and self-blame. Each item is answered on a 1-7 Likert scale. The PTCI has demonstrated excellent internal consistency (α = .97), good test-retest reliability (r = .74), as well as good convergent validity with other measures of beliefs/blame and good discriminant validity in identifying those with and without PTSD. A brief version of this measure will be used.

Daily Spiritual Experience Scale (DSES; Underwood & Teresi, 2002). The DESES has shown a high level of reliability and construct validity across a number of studies. Cronbach's alphas for the scale in English and subsequent translations have been consistently high (e.g., .89 or above). Test-retest over two days has yielded a Pearson correlation of .85 (Underwood, 2002). Additional studies on translated forms have shown good test-retest reliability and a single factor structure (Bailly & Roussiau, 2010; Ng et al., 2009). A 6-item short-form of the scale has been developed and found to be highly correlated with the longer version. We will use an adaptation of the 6-item form.

Social Connectedness (Lee & Robbins, 1995). To date, no measures of social connectedness or belongingness have been constructed for use in refugee research or for those of Islamic faith. We will adapt and utilize a measure used in the broader literature. The original form of this scale has demonstrated high internal consistency of .91, and test-retest correlations have shown to be strong over a 2-week period (r = .96). Cross-validation using confirmatory factor analysis showed an incremental fit index greater than .90.

Transgression-Related Interpersonal Motivations (TRIM-R; McCullough et al., 1998). This measure assesses forgiveness using 5-point anchors, responses ranged from *strongly disagree* to *strongly agree* for each item. The revenge subscale will be used. The TRIM-R shows appropriate variability in test-retest reliability over time (three weeks: r = .83; eight weeks: r = .44) and good construct validity (McCullough et al., 1998).

D. Procedures

Male and female lay leaders will be selected based on being a community leader, having a heart for healing and reconciliation in the Somali community, and willingness to later train additional group leaders. Lay leaders will be able to speak and read in both Somali and English. All lay leaders will sign contracts to be individual investigators affiliated with their respective universities. Lay leaders will attend two, 4-hour training sessions (approximately 6 hours actual training, allowing time for arrival and prayer times), where they will receive the manual and an overview of the program, review and practice components of group sessions, and identify potential issues and problem solve. Lay leaders will be volunteers.

Group participants will be recruited through flyers and word of mouth by the lay leaders, community and faith leaders, and self-referral, focusing on those in need of trauma healing based on their report of re-experiencing and/or avoidance of traumatic events. All participants will sign informed consent forms, in either Somali or English depending on their language preference. Randomization to active or waitlist will be conducted using a 1:1 ratio, allowing for individuals or clusters of up to three individuals being randomized as a unit. Questionnaires will be completed prior to the start of group (Session 1), middle (Session 3), and end (Session 6), with a three-month follow up for individuals in the RCT using a Qualtrics link. Participants will receive fifty dollars at the three-month follow up. Groups will meet weekly for 2 hours, with length of time varying depending on breaks for prayer. After each group, lay leaders will meet briefly with a clinical supervisor from the investigational team, either in person or on phone/online, for a discussion of the session, consultation on content or clinical issues that arose during session, and any supervisory notes. Once the six weekly sessions have been completed, a group social event will be held as a closure ceremony and to facilitate community reconciliation. The content of each group session is clearly delineated in the Islamic Trauma Healing manual.

E. Quality Control

Train the Trainers. Lay leaders will be trained in the “train the trainer” model. This involves experts first training a cohort of lay leaders in methods and techniques of the program in two 4-hour sessions. These sessions include didactic components and competence training (McHugh & Barlow, 2010) such as practice leading sections of the manual and role-plays. The first set of groups (6 sessions) will be led by lay leaders and supervised directly by experts, as outlined in the manual. After delivering the intervention, the first cohort will be trained to pass on skills to the next cohort of lay leaders. The first cohort will be responsible for leading training (two, 4-hour sessions) following content they learned in the initial training and supported by the study team. Subsequently, new lay leaders from the second cohort will co-lead groups with an experienced lay leader from the first cohort. Experts remain as consultants. Handing over training to lay leaders not only increases financial sustainability but also enhances appropriateness and increases disseminatability (e.g., can later be conducted in Somali to reduce requirement for lay leaders to be bilingual).

Fidelity and Clinical Supervision. Following each group session, group leaders will complete a session checklist, designed to be a fidelity checklist with behaviorally specific descriptions of all components of a given session. Group leaders check yes/no as to whether or not they covered the specific component (e.g., “reflected on talking to Allah”), and then record approximately how many minutes they spent with group members on that particular component. These are printed in the manual so all lay leaders will have access to the lists. Clinical supervisors will review these checklists, either in person when meeting face-to-face, or by asking group leaders to read through the checklist when conducting supervision over the phone or online. Clinical supervisors will complete each checklist independently based on report by lay leaders and include the checklist as part of the written clinical note following supervision. Use of these checklists allows for consistent monitoring of how closely group leaders are adhering to the manual, ensures comprehensive records are kept in the case of remote supervision, and serves as a platform for providing feedback to group leaders when supervisors notice drift from protocol. Lay leaders will learn during training about the requirements of clinical supervision.

F. Power Analysis

We estimated sample size based primarily on Specific Aim 1. We estimated power to detect effects using G*Power and Optimal Design (for multilevel data), with $\alpha = .05$ and power $(1 - \beta) = .80$, completing 10,000 replications per analysis. With a conservative estimated dropout of 20% across conditions, though no dropout occurred in our pilot work, we will have power to detect moderate to large effects of condition on outcomes at post-treatment ($d = 0.72$) with 30 per group, and ($f^2 = 0.28$) with generalized linear models (GLM),

both large effects. For repeated measures analyses, with 4 data points, 30 per group with 20% missing data, we will be able to detect medium effects (d = 0.60) on change over time. These effect sizes are well below the observed effects in our pilot work (g = 0.76 to g = 3.22). For Specific Aim 2, the same power calculations for Aim 1 apply; however, tests of indirect effects are exploratory, we will be powered to detect a moderate indirect effect ($a*b$ = 0.25), using the same constraints as above. To balance feasibility and ability to detect meaningful effects, we will recruit 30 individuals per treatment group

G. Analytic Strategy

Data will be screened for accuracy, missing values, and fit between the data and the assumptions of the general linear and linear mixed-model analysis (e.g., linearity, multivariate normality, independence of errors; Tabachnick & Fidell, 2007). Data transformations or model respecifications will be performed for any violated assumptions. We will implement pattern-mixture models to examine whether the missingness can be considered ignorable. For non-ignorable missing data, values will be multiply imputed and data recombined using Rubin's rules (Rubin, 1987) in the mixed model program. We will examine associations between primary dependent variables and factors such as gender, age, time since trauma, etc. and make appropriate analytic adjustments. We will test all covariate by predictor interactions to ensure our main effects are independent of the level of these putative covariates; these tests of effect modification are easily accommodated. However, we are underpowered to assess these interactions. To account for subjects being nested within groups in the treatment condition, we will co-vary treatment group using dummy codes in all analyses, as linear mixed models are difficult to estimate when the number of clusters is very small (Snijders, 2005).

Specific Aim 1: Therapeutic Effects

Hypothesis: Those in Islamic Trauma Healing will show a greater reduction of PTSD symptoms and related psychopathology than those in the waitlist condition (WL).

Predictor Variables: Predictor is treatment (Islamic Trauma Healing vs. WL)

Dependent Variables: PTSD severity (PS-SR-5, primary outcome), depression (PHQ-9), somatic symptoms (PHQ-15), and well-being (WHO-5) will be measured.

Analyses: Because of the presence of clustering (participants in groups, observations over time, and participants in random assignment clusters), we will use generalize linear mixed models (GLMMs), which can account for the presence of clustering in data with varying distributional forms. We will compare pre-post GLMMs with those using data from all four time points to estimate the effects of treatment on both the level of the outcome at post-treatment, as well as change over time. To account for clustering (in treatment groups and random assignment clusters), we will include random intercepts for both treatment group and cluster (for those choosing to be randomized in a cluster). Because the waitlist control and non-clustered individuals will *not* be in a cluster, we will use the method outline by Sterba and Bauer (2008) to model random intercepts for *only* those in treatment or a cluster, and test whether modeling residuals as a function of clustering status appropriately models heteroskedasticity of residuals across clustering status. To test models of change over time, these models would be extended to three level models, with random intercepts and random coefficients for time-varying covariates (e.g., time) specified at Level 2 if the model fit is better than a fixed effects model.

Dropout. We will compare the percent of those who drop out of Islamic Trauma Healing to WL using a chi-square test. Reasons for dropout will be documented and compared across groups. Psychopathology and functioning of dropouts will be compared, using chi-square or t-tests, as appropriate.

Satisfaction. Means and standard deviation of satisfaction (CSQ) will be reported. A comparison to WL does not make sense, as there are no services with which to compare.

Specific Aim 2: Potential Mediators Change with Active Intervention

Hypothesis: Those in Islamic Trauma Healing will show a greater reduction of negative beliefs (PTCI), connectedness (SC; DSES), and/or forgiveness (TRIM-R) than those in the waitlist condition (WL).

Predictor Variables: Predictor is treatment (Islamic Trauma Healing vs. WL)

Dependent Variables: Negative beliefs (PTCI), connectedness (SC; DSES), and forgiveness (TRIM-R) will be measured.

Analyses: We will conduct similar analyses to Aim 1, using both GLMs and GLMMs to obtain effect size estimates. In the GLMs, treatment will predict negative beliefs, connectedness, and forgiveness from baseline to post-treatment (controlling for baseline levels). GLMMs will be conducted as above, and treatment will be used to predict post-treatment levels and change over time in the dependent variables, to estimate how groups

differ in their rate of symptom reduction over the course of treatment.

Exploratory Hypothesis. Changes in negative beliefs (PTCI), connectedness (SC; DSES), and/or forgiveness (TRIM-R) will mediate the relationship between treatment condition and changes in PTSD and related symptoms. For the GLMs, we will predict Session 3 mediators from treatment (a path) and follow-up outcomes from the mediator (b path) controlling for treatment and baseline levels. Mediation will be tested using the product of coefficients method with bootstrapped standard errors (MacKinnon et al., 2009). In the GLMMs, we will conduct a 2-1-1 mediation, predicting the mediator at all time points from treatment (2-1), and the outcome at all time points from the mediator (1-1), controlling for treatment. This will be based on Bauer et al. (2006), generally following the product of coefficients method. It is recognized that this is underpowered.
