# Supplementary material for: Lay-Led Intervention for War and Refugee Trauma: A Randomized Clinical Trial
Source: JAMA Netw Open. 2024 Aug 26;7(8):e2429661. doi: 10.1001/jamanetworkopen.2024.29661 (PMC11423170; doi:10.1001/jamanetworkopen.2024.29661)
Supplement: Supplement 2. — Nonauthor Collaborators. The Islamic Trauma Healing Clinical Team [file jamanetwopen-e2429661-s002.pdf]

Supplemental Online Content: Nonauthor Collaborators
*First name, last name, and suffix (if applicable) are required and will appear in PubMed.

***Group Name(s): Islamic Trauma Healing (ITH) Clinical Team**

***First Name and Middle Initial(s)**	***Last Name**	***Suffix (eg, Jr, III)**	Academic Degrees	Institution	Location (city, state/province, country)	Role or Contribution, eg, chair, principal investigator	Group (if more than 1 Group listed in the byline) and/or Subgroup (eg, Steering Committee)
Hana	Ali			Somali Family Safety Task Force	Seattle, WA	community leader	Islamic Trauma Healing (ITH) Clinical Team
Yasmin	Ibrahim			Somali Family Safety Task Force	Seattle, WA	community leader	Islamic Trauma Healing (ITH) Clinical Team
Abdi	Ismail			Somali Family Safety Task Force	Seattle, WA	community leader	Islamic Trauma Healing (ITH) Clinical Team
Safiya	Omar			Somali Family Safety Task Force	Seattle, WA	community leader	Islamic Trauma Healing (ITH) Clinical Team
Mohamed	Roble		M.S.	Somali Family Safety Task Force	Seattle, WA	community leader	Islamic Trauma Healing (ITH) Clinical Team
Warsame	Roble			Somali Family Safety Task Force	Seattle, WA	community leader	Islamic Trauma Healing (ITH) Clinical Team
Nafiso	Samatar			Somali Family Safety Task Force	Seattle, WA	community leader	Islamic Trauma Healing (ITH) Clinical Team
Mohammed	Alsubaie		Ph.D.	Seattle Pacific University	Seattle, WA	master's/ doctoral level supervising clinician	Islamic Trauma Healing (ITH) Clinical Team
Michael	Dolezal		Ph.D.	Seattle Pacific University	Seattle, WA	master's/ doctoral level supervising clinician	Islamic Trauma Healing (ITH) Clinical Team
Alexandra	Klein		Ph.D.	Case Western Reserve University	Cleveland, OH	master's/ doctoral level supervising clinician	Islamic Trauma Healing (ITH) Clinical Team
Emma	PeConga			University of Washington	Seattle, WA	master's/ doctoral level supervising clinician	Islamic Trauma Healing (ITH) Clinical Team
Peter	Rosencrans		Ph.D.	University of Washington	Seattle, WA	master's/ doctoral level supervising clinician	Islamic Trauma Healing (ITH) Clinical Team

Supplemental Online Content: Nonauthor Collaborators
*First name, last name, and suffix (if applicable) are required and will appear in PubMed.

***First Name and Middle Initial(s)**	***Last Name**	***Suffix (eg, Jr, III)**	Academic Degrees	Institution	Location (city, state/province, country)	Role or Contribution, eg, chair, principal investigator	Group (if more than 1 Group listed in the byline) and/or Subgroup (eg, Steering Committee)
Rosemary	Walker		Ph.D.	University of Washington	Seattle, WA	master's/ doctoral level supervising clinician	Islamic Trauma Healing (ITH) Clinical Team
Alexandra	Bowling			Case Western Reserve University	Cleveland, OH	research support staff	Islamic Trauma Healing (ITH) Clinical Team
Ashleigh	Holloway			University of Washington	Seattle, WA	research support staff	Islamic Trauma Healing (ITH) Clinical Team
Ifrah	Sheikh		MSc, MA	Georgia State University	Atlanta, GA	research support staff	Islamic Trauma Healing (ITH) Clinical Team
Eesha	Ali		BA	University of Washington	Seattle, WA	research support	Non-Author Contributer
