# Supplementary material for: Lay-Led Intervention for War and Refugee Trauma: A Randomized Clinical Trial
Source: JAMA Netw Open. 2024 Aug 26;7(8):e2429661. doi: 10.1001/jamanetworkopen.2024.29661 (PMC11423170; doi:10.1001/jamanetworkopen.2024.29661)
Supplement: Supplement 3. — Data Sharing Statement [file jamanetwopen-e2429661-s003.pdf]

Data Sharing Statement

Zoellner. Lay-Led Intervention for War And Refugee Trauma. *JAMA Netw Open*. Published August 26, 2024. doi:10.1001/jamanetworkopen.2024.29661

Data
Data available: Yes
Data types: Deidentified participant data
How to access data: The data for the trial is available through the National Institute of Mental Health Data Archive (#52756): https://nda.nih.gov
When available: With publication

Supporting Documents
Document types: Statistical/analytic code
How to access documents: Analytic code available upon request from kingkm@uw.edu
When available: With publication

Additional Information
Who can access the data: Per NIMH Data Archive protocol.
Types of analyses: Per NIMH Data Archive protocol.
Mechanisms of data availability: Per NIMH Data Archive protocol.
Any additional restrictions: Per NIMH Data Archive protocol.
